# Function of RasGRP3 in the formation and progression of human breast cancer

**DOI:** 10.1186/1476-4598-13-96

**Published:** 2014-04-29

**Authors:** Zsuzsanna Nagy, Ilona Kovács, Miklós Török, Dezső Tóth, György Vereb, Krisztina Buzás, István Juhász, Peter M Blumberg, Tamás Bíró, Gabriella Czifra

**Affiliations:** 1 DE-MTA “Lendület” Cellular Physiology Research Group, Department of Physiology, University of Debrecen, Medical and Health Science Center, Research Center for Molecular Medicine, Nagyerdei krt. 98, PO Box 22, Debrecen H-4032, Hungary; 2 Department of Pathology, Gyula Kenézy Hospital, Debrecen, Hungary; 3 Department of Surgery, Gyula Kenézy Hospital, Debrecen, Hungary; 4 Department of Biophysics and Cell Biology, University of Debrecen, Medical and Health Science Center, Faculty of Medicine, Debrecen, Hungary; 5 Institute of Biochemistry, Biological Research Center of the Hungarian Academy of Sciences, Szeged, Hungary; 6 Department of Dermatology, University of Debrecen, Medical and Health Science Center, Debrecen, Hungary; 7 Laboratory of Cancer Biology and Genetics Center for Cancer Research, National Cancer Institute, Bethesda, MD, USA

**Keywords:** Ras activator, RasGRP3, Human breast cancer, Chemotherapeutic resistance, Tamoxifen, Trastuzumab, Tumorigenesis, EGF, IGF-I, Signaling pathways

## Abstract

**Introduction:**

Ras guanine nucleotide exchange factors (RasGEFs) mediate the activation of the Ras signaling pathway that is over activated in many human cancers. The RasGRP3, an activator of H-Ras and R-Ras protein exerts oncogenic effects and the overexpression of the protein is observed in numerous malignant cancer types. Here, we investigated the putative alteration of expression and potential function of RasGRP3 in the formation and progression of human breast cancer.

**Methods:**

The RasGRP3 and phosphoRasGRP3 expressions were examined in human invasive ductal adenocarcinoma derived samples and cell lines (BT-474, JIMT-1, MCF7, SK-BR-3, MDA-MB-453, T-47D) both in mRNA (Q-PCR) and protein (Western blot; immunohistochemistry) levels. To explore the biological function of the protein, RasGRP3 knockdown cultures were established. To assess the role of RasGRP3 in the viability of cells, annexin-V/PI staining and MitoProbe™ DilC1 (5) assay were performed. To clarify the function of the protein in cell proliferation and in the development of chemotherapeutic resistance, CyQuant assay was performed. To observe the RasGRP3 function in tumor formation, the Severe combined immunodeficiency (SCID) mouse model was used. To investigate the role of the protein in Ras-related signaling Q-PCR and Western blot experiments were performed.

**Results:**

RasGRP3 expression was elevated in human breast tumor tissue samples as well as in multiple human breast cancer cell lines. Down-regulation of RasGRP3 expression in breast cancer cells decreased cell proliferation, induced apoptosis in MCF7 cells, and sensitized T-47D cells to the action of drugs Tamoxifen and trastuzumab (Herceptin). Gene silencing of RasGRP3 reduced tumor formation in mouse xenografts as well. Inhibition of RasGRP3 expression also reduced Akt, ERK1/2 and estrogen receptor alpha phosphorylation downstream from IGF-I insulin like growth factor-I (IGF-I) or epidermal growth factor (EGF) stimulation confirming the functional role of RasGRP3 in the altered behavior of these cells.

**Conclusions:**

Taken together, our results suggest that the Ras activator RasGRP3 may have a role in the pathological behavior of breast cancer cells and may constitute a therapeutic target for human breast cancer.

## Introduction

The activation of Ras family members is conveyed by specific upstream regulators such as e.g. guanine nucleotide exchange factors (RasGEF) [1]. Reflecting their significance, genetic loss of these factors and their functions was shown to result in similar effects to those induced by the loss of the Ras proteins themselves [2,3]. In 1998, Ebinu et al. have introduced a new RasGEF called Ras Guanyl nucleotide Releasing Peptide or RasGRP [4]. A unique feature of RasGRPs is the presence of a C1 domain serving as a binding site for the endogenous signaling molecule diacylglycerol (DAG) and its exogenous counterparts, the phorbol esters. As RasGRPs are regulated directly by binding DAG [5,6], they act as mediators for the a multitude of receptor-coupled mechanisms (induced e.g. by numerous growth factors) that activate phospholipase C and the related signal transduction machineries [7,8]. RasGRP3 is one of the four members of RasGRP family; it was shown to activate H-Ras and R-Ras [4,9].

The presence and activity of the Ras-related subcellular processes and the induced biological responses are one of the key determinants in malignant cell transformation and progression. Efforts to identify the nature and role of the Ras-related intracellular signalization pathways have recently suggested that not only the downstream mediators but also the upstream regulators of Ras might play a role on these mechanisms. In perfect agreement with this proposal, members of RasGRP family are suggested to function as oncogenes in multiple cancers [10]. Indeed, RasGRP3 is highly expressed in human Burkitt’s lymphoma, human pre-B-cell leukemia, and natural killer-like T-cell leukemia. Of further importance, we have recently shown that RasGRP3 is centrally involved in the regulation of cell proliferation, survival, migration and tumor formation of human prostate carcinoma cells and several melanoma cell lines as well as in the malignant transformation of human melanocytes [11-13].

These findings which implicate pro-oncogenic effect of the protein and, furthermore, the fact that Ras was found is chronically activated in breast carcinoma cells that lack mutated *ras *[14], have motivated us to assess the expression and function of RasGRP3 in breast derived invasive ductal adenocarcinoma, a malignancy characterized by a high tendency to produce metastatic tumors [15]. In the present study, we provide evidence that expressions of RasGRP3 and its active form (phosphoRasGRP3) are highly elevated in human breast cancer samples and that it is also present in a primary and several metastatic human breast cancer cell lines. Moreover, we also show that RasGRP3 contributes to proliferation, survival, chemotherapeutic resistance and in vivo tumor growth of breast carcinoma-derived tumor cell lines.

## Results

### RasGRP3 is elevated in human breast derived ductal adenocarcinoma

**Figure 1:**
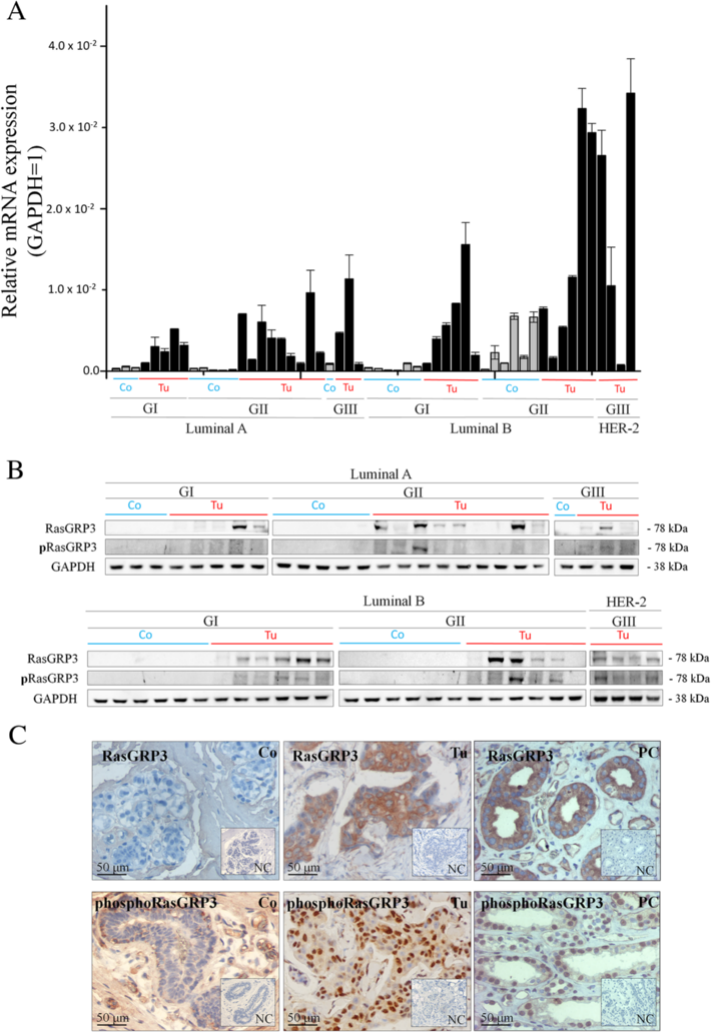
**RasGRP3 is expressed in human breast derived ductal adenocarcinoma. (A)** Q-PCR and **(B)** Western blot analysis of RasGRP3 and phosphoRasGRP3 expressions in human normal breast (Controls; n = 21) and breast derived ductal adenocarcinoma (Tumors; n = 33) with various tumor grades (G1-G3) presented according to the molecular grading of human breast derived ductal adenocarcinoma (Luminal A; Luminal B and HER-2). GAPDH was used as an internal control. The results are representative of three independent experiments for Q-PCR and two independent experiments for Western blot. Values represent the mean ± SEM. **(C)** Representative images of RasGRP3- and phosphoRasGRP3-specific immunreactivity with diaminobenzidine as a chromogen (brown staining) on human normal and breast derived ductal adenocarcinoma sections. Human kidney samples were included as a positive control (PC) [16]. Nuclei were co-stained by Mayer’s Hematoxylin (blue staining). Preabsorption negative control (NC) was used. Images were taken at magnifications 20x. The results are representative of four independent experiments.

First, using Q-PCR (Figure 1A) and Western blot (Figure 1B), we assessed the expression of the RasGRP3 protein and presumably its active form, phosphoRasGRP3, in human breast derived ductal adenocarcinoma (Tu) as well as in normal human breast tissue (Co) samples. We found that the levels of RasGRP3 and phosphoRasGRP3, albeit exhibiting marked inter-individual variations, were significantly higher in the tumor samples compared to the controls. In addition, we also determined the cellular localizations of RasGRP3 and phosphoRasGRP3 by immunohistochemistry in sections prepared from the diseased breast tissues. RasGRP3 was localized in the cytoplasm of the cells whereas the active form exhibited mostly nuclear immunoreactivity (Figure 1C). Human kidney derived samples (9) served as positive controls (PC) in immunohistochemical experiments.

### RasGRP3 is expressed in human breast derived ductal adenocarcinoma cell lines and involved in the regulation of growth of MCF7 and T-47D cells

**Figure 2:**
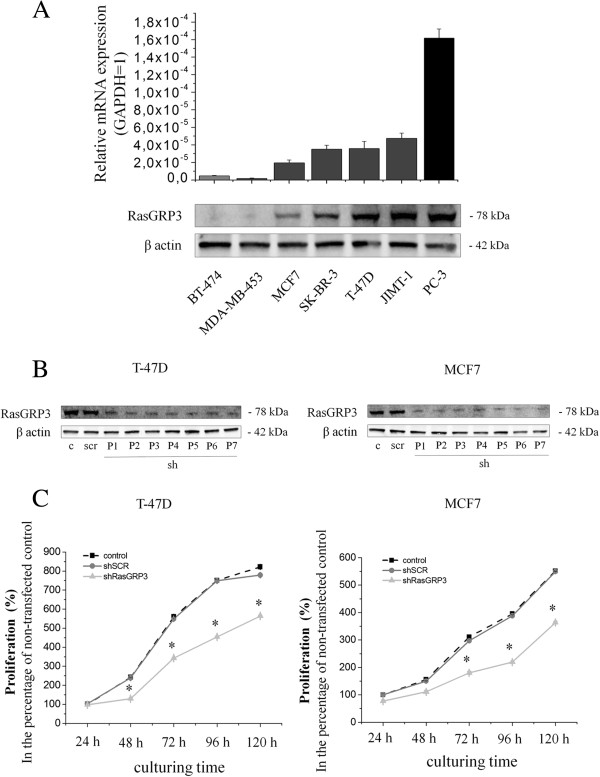
**RasGRP3 is expressed in human breast derived ductal adenocarcinoma cell lines and represses cell proliferation of both MCF7 and T-47D derived cells. (A)** Q-PCR and Western blot analyses of RasGRP3 expression in multiple breast derived ductal adenocarcinoma cell lines. PC-3 cells were included as a positive control [12]. GAPDH was used as an internal control. The results are representative of three independent experiments. Values represent the mean ± SEM. Expression of RasGRP3 was inhibited using a specific shRNA (shRasGRP3). A non-targeting scrambled shRNA (shSCR) was used as a control. **(B)** Confirmation of the extent of suppression of RasGRP3 expression was determined by Western blotting. GAPDH was used as an internal control. P indicates the number of subculturing of the RasGRP3 silenced cells. Results are representative of 2 independent experiments. **(C)** The proliferation of T-47D and MCF7 derived cells was determined using the CyQuant GR cell proliferation assay, with values normalized to the levels of non-transfected control cells. CyQuant assay was conducted every 24 hours. Values represent the mean ± SEM for 2 independent experiments.

We determined the expression of RasGRP3 in six different human breast ductal adenocarcinoma derived cell lines, namely in BT-474, MDA-MB-453, MCF7, SK-BR-3, T-47D and JIMT-1 cells (the characteristics of the cell lines and relevant references are shown in Additional file 1: Table S1). Quantitative PCR and Western blot analyses confirmed the expression of RasGRP3 in all cell lines with barely detectable levels in BT-474 and MDA-MB-453 cells (Figure 2A). For comparison, the relatively high expression of RasGRP3 in the PC-3 prostate adenocarcinoma derived cell line [12] served as a positive control in both assays.

To explore the functionality of RasGRP3, namely the role of the protein in the regulation of proliferation, viability, chemotherapeutic resistance and tumorigenesis we employed the shRNA-interference technique. Namely, the cellular levels of the protein in MCF7 and T-47D cells was suppressed by retroviral vectors expressing shRNAs for RasGRP3 (shRasGRP3) or against a non-targeting scrambled control (shSCR) to achieve a long term (stable) suppression (sequences of the shRNA probes are shown in Additional file 2: Table S2). As determined by confirmatory Western blot analysis, levels of RasGRP3 could be effectively reduced in both MCF7 and T-47D cancer cells whereas in cells expressing shSCR significant modulation of RasGRP3 expression was not observed (Figure 2B).

We assessed the possible alterations in cellular functions of the T-47D and MCF7 derived “RasGRP3-silenced” cells. As revealed by growth curve analysis using fluorimetric CyQUANT cellular proliferation assays from days 0–5 of culturing (Figure 2C), down-regulation of RasGRP3 expression resulted in a significant suppression of cell growth in both cell lines compared to the proliferation of cells bearing the non-targeting shSCR.

### RasGRP3 is involved in the regulation of survival of MCF7 tumor cells

**Figure 3:**
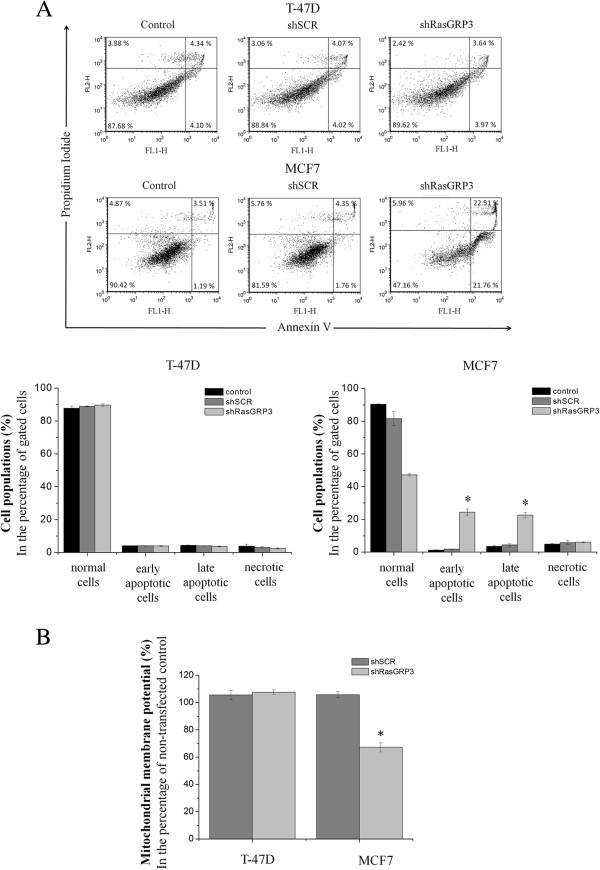
**Down-regulation of RasGRP3 expression induces apoptosis in MCF7 derived cells. (A)** Representative images of MCF7 and T-47D derived cells stained with Annexin-V and PI and analyzed by flow cytometry. Data were evaluated with FSC Express softwer and presented in the percentage of gated cells. Values represent the mean ± SEM for three independent experiments. **(B)** Assessment of mitochondrial membrane potentials of MCF7 and T-47D derived cells by fluorimetric DilC1 (5) assay with values normalized to the levels of non-transfected control cells. Values represent the mean ± SEM for four independent experiments.

Furthermore, as determined by flow cytometric analysis following annexin-V/PI labeling, inhibition of RasGRP3 expression induced apoptosis in MCF7 cells but not in the T-47D cell line (Figure 3A). To further support the role of RasGRP3 in the regulation of apoptosis a quantitative fluorimetric MitoProbe™ DilC1 (5) assay was performed. A reduction in the mitochondrial transmembrane potential is one of the earliest markers of apoptosis [16,17]. MitoProbe DilC_1_ (5) is a mitochondrial membrane potential sensitive dye which accumulates in the mitochondria in cells with active membrane potential. The staining intensity decreases in cells with disrupt mitochondrial membrane potential. We found that RasGRP3 silencing significantly decreased mitochondrial membrane potential (Figure 3B), in MCF7 cells, while no significant change was observed in T-47D cells.

### RasGRP3 expression contributes to resistance to Tamoxifen and Herceptin in the MCF7 and T-47D breast cancer tumor cells

**Figure 4:**
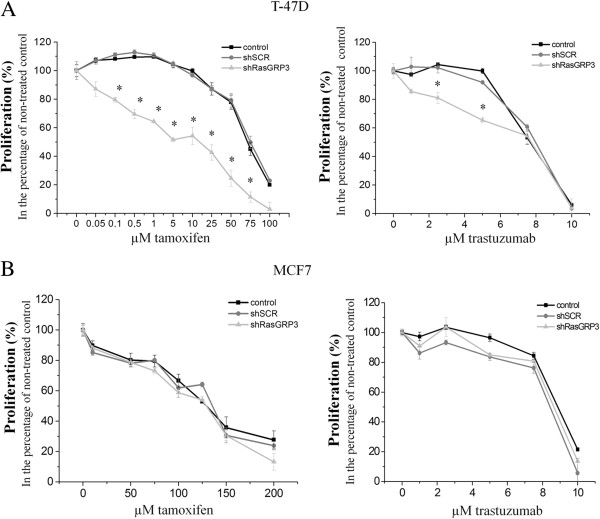
**Down-regulation of RasGRP3 increases the sensitivity to tamoxifen and trastuzumab of the T-47D cell line.** The indicated MCF7 and T-47D derived cell lines were seeded at a density of 1 x 10^4^ cells/ well and treated with tamoxifen **(A)** and trastuzumab **(B)** (tamoxifen: MCF7 cells: 0–200 μM; T-47D cells: 0–100 μM; trastuzumab: both MCF7 and T-47D cells: 0–10 μM). After 72 hours of incubation cell proliferation was determined using the CyQuant GR cell proliferation assay. The results were normalized to the levels of untreated cells. Values represent the mean ± SEM of 2 independent experiments.

We also investigated the sensitivities of “RasGRP3-silenced” MCF7 and T-47D cells to tamoxifen (Tamoxifen) used in endocrine therapy and to the chemotherapeutic drug trastuzumab (Herceptin) [18,19]. Down-regulation of RasGRP3 increased the sensitivity of T-47D cells to the growth-inhibitory actions of both tamoxifen and trastuzumab; i.e. lower concentration induced comparable effects to those found with higher concentrations on cells expressing the control shSCR (Figure 4A). Interestingly, suppression of RasGRP3 levels did not affect the sensitivity of the MCF7 cells to the actions of the drugs (Figure 4B).

### Down regulation of RasGRP3 suppressed xenograft tumor formation

**Figure 5:**
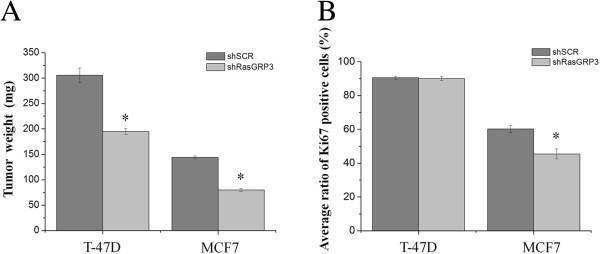
**Suppression of RasGRP3 expression inhibits xenograft tumor growth of both MCF7 and T-47D cells.** SCID mice were injected subcutaneously with 4x10^6^ cells of the indicated MCF7 and T-47D derived cells (n = 4 mice for each group). The animals were sacrificed 12 weeks after injection, and the tumors were excised, weighted **(A)** and processed for immunohystochemical analysis. Values represent the mean ± SEM of four independent experiments. **(B)** Digitalized images of Ki67-specific immunreactivity (Additional file 3: Figure S1 and Additonal file 4: Figure S2) obtained on sections prepared from tumors developed by MCF7 and T-47D derived cells were analysed using Image J image analysis softwer. In every each section the number of cells were counted at 5 randomly placed, equal areas of interest (AOI) and the average of Ki67 imunnopositive cells of the 5 AOI was defined. Ratios: in T-47D shSCR n = 1213/1098; in T-47D shRasGRP3 n = 1216/1096; in MCF7 shSCR n = 1268/757; in MCF7 shRasGRP3 n = 1112/553. The numbers represent total cell number/Ki67 positive cells ratios. Values represent the mean ± SEM.

To assess furthermore the role of RasGRP3 in *in vivo* tumor formation, we employed the SCID mouse xenograft model in which tumors were induced by MCF7 and T-47D cells expressing either shRasGRP3 or shSCR. In both cell lines, down-regulation of RasGRP3 resulted in a marked reduction in tumor growth (Additional file 3: Figure S1 and Additional file 4: Figure S2) as measured by weight of excised tumors in comparison of those induced by the shSCR-expressing cells (Figure 5A). These differential features of the various cells on tumorigenesis were also proven by immunohistochemical analysis of the expression of the proliferation marker Ki67. In tumors induced by RasGRP3-silenced MCF7 cells the number of Ki67 positive cell was significantly less than in the control shSCR-expressing ones (Figure 5B and Additional file 3: Figure S1 and Additional file 4: Figure S2). Interestingly, despite the significantly less tumor size, statistical analysis did not reveal differences in the Ki67 positive cell number in the case of the T-47D cells.

### RasGRP3 is involved in growth factor-induced Akt, ERK1/2 and estrogen receptor activation

**Figure 6:**
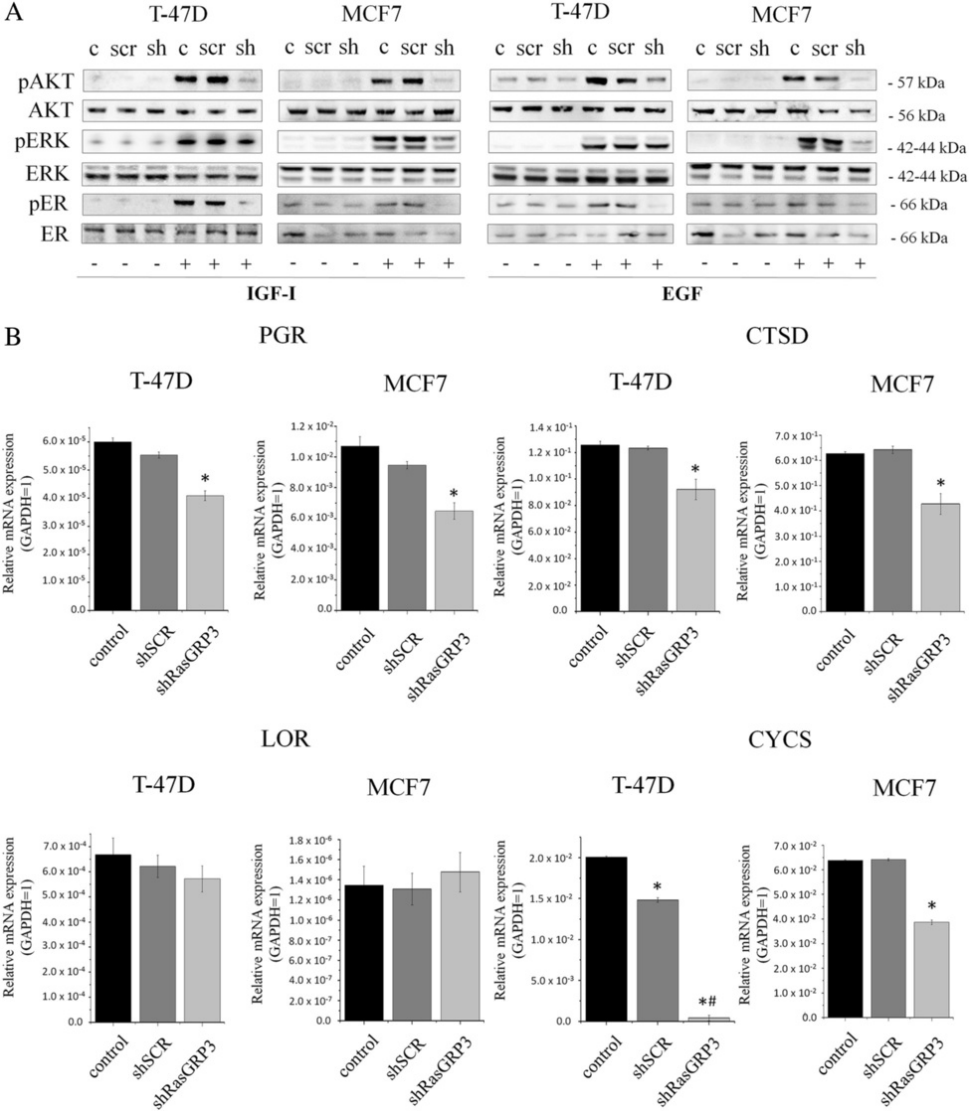
**Effects of down-regulation of RasGRP3 on the Ras signaling pathway I.** RasGRP3 is involved in IGF-I and EGF **(A)** dependent Akt, ERK and ERα phosphorilation. RasGRP3 knockdown cell lines created from MCF7 and T-47D cells were treated with or without IGF-I (100 pg/ml) and EGF (100 pg/ml) as indicated. Akt and phosphorylated Akt, ERK and phosphorylated ERK, ERα and phosphorylated ERα were detected by immunoblotting of cell lysates. Levels of total Akt, ERK and ERα were used as control. All results were representative of 2 independent experiments. **(B)** Q-PCR analyses of ERα-regulated genes, namely progesterone receptor (PGR), cathepsin D (CTSD), cytochrome C (CYCS) and loricrin (LOR). GAPDH was used as an internal control. The results are representative of three independent experiments. Values represent the mean ± SEM. In case of T-47D cells loricrin expression *indicates significant difference compared to non-transfected control cells, while ^#^indicates significant difference compared to scrambled control cells.

Growth factors such as insulin like growth factor-I (IGF-I) and epidermal growth factor (EGF) represent important signaling molecules in breast cancer [20,21]. In the final stage of our experiments, we therefore evaluated the role of RasGRP3 in modulating the IGF-I and EGF induced activation of the Ras signaling pathway in both MCF7 and T-47D cells. Cells were treated with IGF-I and EGF (Figure 6A) as indicated, and the activation of the possibly most important downstream molecules related to Ras, ERK 1/2 and Akt kinases were examined by Western blot. We confirmed that in both cell lines the down-regulation of RasGRP3 reduced the IGF-I and EGF-induced ERK 1/2 and Akt phosphorylation to the basal level (Figure 6A and B). Consistent with the central role of Ras signaling pathway in the activation of estrogen receptor alpha (ERα), decreasing the level of pERK 1/2 and pAkt led to a reduction in ERα phosphorylation (Figure 6A).

According to our results MCF7 cells were more sensitive to the growth factors than T-47D cells and the onset of phosphorilation events was different, with MCF7 derived “RasGRP3-silenced” cells appearing to respond to growth factors induced Akt and ERK1/2 down-regulation earlier. In case of IGF-I application (Additional file 5: Figure S3) in MCF7 cells, pAKT was affected in 5–30 min, while pAKT is affected only at 30–40 min in T-47D cells. A marked reduction in ERK ½ activation can be observed in both cells at 5 and 10 min, which is still present at 20 and 30 mins in T-47D cells. A decrease in ERα phosphorilation can be noticed in MCF7 cells earlier, but the down-regulation is most affected in T-47D cells. In case of EGF treatment (Additional file 6: Figure S4) the RasGRP3 mediated down-regulation of Akt (5–40 min) and ERK ½ (5–20 min) activation was more effective in MCF7 cells compared to T-47D, in which pAkt is affected only at 30 min and there is no significant change in ERK ½ activation. However T-47D cells show a significant decrease in ERα activation (20–30 min) compared to MCF7 cells, in which pERα is affected only at 20 min. Importantly, down regulation of RasGRP3 had no measurable effect on basal Akt, ERK1/2 and ERα phosphorylation and on the total Akt, ERK1/2 and ERα expression levels.

To validate further a role for RasGRP3 in the modulation of the activation of ERα we examined the effect of RasGRP3 gene-silencing on the expression of several ERα-regulated genes [22], namely progesterone receptor (PGR), cathepsin D (CTSD), cytochrome C (CYCS) and loricrin (LOR) determined by Q-PCR. Except loricrin we found a significant reduction in the expression of these genes in “RasGRP3-silenced” MCF7 and T-47D cells (Figure 6B). In case of T-47D cells a significant decrease was observed in shSCR cells in their cytochrome C expression compared to the non-treated cells, but in shRasGRP3 cells this decrease was more expressive.

### RasGRP3 modulates the expression of IGF-I and EGF receptors in MCF7 cells

**Figure 7:**
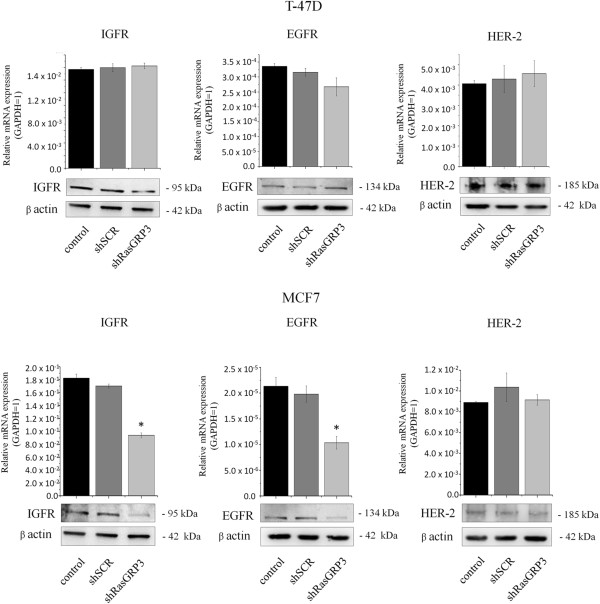
**Effects of down-regulation of RasGRP3 on the Ras signaling pathway II.** IGF1R, EGFR and HER-2 expressions were detected by Q-PCR and immunoblotting of RasGRP3 knockdown cell lines created from MCF7 and T-47D cells. GAPDH (Q-PCR) and β actin (Western Blot) were used as internal controls. The results are representative of three (Q-PCR) or two (Western blot) independent experiments. Values represent the mean ± SEM.

To further clarify the role of RasGRP3 on the activation of the downstream signaling molecules, the effect of RasGRP3 inhibition on the expression of upstream IGF-I, EGF, and Human Epidermal Growth Factor Receptor 2 (HER-2) receptors was investigated (Figure 7). Suppression of RasGRP3 levels decreased the expression of both IGF-I and EGF receptors in MCF7 cells, whereas it had no effect on the receptor expression of T-47D cells. RasGRP3 down-regulation did not modify the expression of HER-2 receptor of the cells.

## Discussion

Activation of Ras signaling has long been recognized to be important for tumorigenesis and progression of cancer cells [23,24]. Moreover, it was also suggested that Ras can be “chronically overactivated” by various upstream signaling elements [15] such as e.g. RasGRPs [1].

Previous studies have clearly identified the existence of RasGRP3 on human Burkitt’s lymphoma, pre-B-cell leukemia, natural killer-like T-cell leukemia, metastatic prostate cancer and malignant melanoma [11-13]. Using Q-PCR and tissue microarray it was proved that RasGRP3 is overexpressed in prostate tumor and melanoma samples. However, to our best knowledge, we are the first to describe and quantitatively asses the expression of RasGRP3 and its active form on human breast ductal adenocarcinoma cells. Indeed, mutually complementary Q-PCR, Western blot and immunohistochemical analyses revealed that the expression of RasGRP3 and the active phosphoRasGRP3 are elevated in numerous human breast tumor samples as well as in multiple breast derived ductal adenocarcinoma cell lines. We have also presented that the relative level of RasGRP3 and phosphoRasGRP3 expressions in the tumor tissues was markedly higher compared to the normal tissues. We also presented the expression of RasGRP3 in breast cancer derived cell lines. Of further importance, we are the first to show that the subcellular localization pattern of RasGRP3 and its active form are markedly different; i.e., RasGRP3 is localized mostly in the cytoplasm whereas phosphoRasGRP3 showed prominent nuclear staining in the breast cancer cells. RasGRP3 is a high-affinity target for the second messenger diacylglycerol (DAG), its analogues and phorbol-esters (PMA) regulating their translocation and exchange factor activity [5,6]. As previously shown RasGRP3 localized mainly in the cytosol [6]. 1,2-dioctanoyl-sn-glycerol (DOG), a membrane-permeable analogue of DAG and phorbol ester PMA also induced RasGRP3 redistribution to the perinuclear region and, to a lesser extent, to the plasma membrane [6]. The localization to the plasma membrane is relevant for the activation of Ras [25]. There are several examples of RasGEFs for Ras that are recruited to the plasma membrane with activation, such as SOS and RasGRF2 [26,27]. Localization of RasGRP3 in the nucleus has not been reported before, and its significance is puzzling. However, forms of H-Ras and Rap1, which are both potential targets of RasGRP3, have been found in the nuclei of *N*-nitrosodiethylamine-induced liver tumor cells and squamous cell carcinoma lines, respectively [28,29]. In fact, subcellular redistribution in response to DAG or its analogs is one of the hallmarks of activation of RasGRPs, the interaction of the protein with specific intracellular proteins can also participate in the translocation events. The role of an adaptor protein RACK has been shown in the case of a well known DAG receptor protein kinase C [30]. Dynein light chain 1 (DLC1), a component of the cytoplasmic dynein complex is a novel RasGRP3-interacting protein participating in the subcellular localization of RasGRP3 [31]. DLC1 is not only involved in cytoskeleton-mediated motility and intracellular transport events [32] but was also reported responsible to regulate the nuclear localization of certain proteins, namely p53 and brain-enriched protein PMES-2 [33,34]. DLC1 also participates in the transactivation functions of ERα [35] and interacts with nuclear transcription factors in fission yeast [36]. As DLC1 is often overexpressed in breast cancer [37] it is interesting to note that DAG induced DLC1–RasGRP3 interaction may act as a chaperone for nuclear translocation of RasGRP3 in breast cancer cells suggesting a mechanistic role for RasGRP3 in supporting and amplifying growth factor or Ras initiated cellular responses in breast cancer cells. Obviously further investigations are needed to support this theory.

Both *in vitro* and *in vivo* studies were carried out to define the exact functional role of RasGRP3 and the coupled intracellular signaling using RasGRP3 gene silencing in ductal adenocarcinoma derived MCF7 and T-47D cell lines. Similar to those described in prostate carcinoma and melanoma [12,13], down-regulation of RasGRP3 inhibited cell proliferation of both cell lines and induced apoptosis in MCF7 cells. Among the growth factor signaling molecules, the major pathway implicated in supporting survival is the phosphatidylinositide 3-kinase (PI3K) pathway. Studies indicate that the protective effect of PI3K is mediated primarily by Akt [38]. In addition, inhibition of Akt signalling can induce apoptosis in some human cancer cell lines [39,40]. Because there are multiple downstream effectors of Akt, it is not completely clear how the survival signal is transduced. Given the pivotal role of BCL-2 family in the regulation of apoptosis previous reports have shown that Akt delivers antiapoptotic survival signals by phosphorilating Bad therefore inhibits apoptosis by maintaining Bcl-x_L_ function and preventing cytochrome c release from mitochondria. We found the inhibition of RasGRP3 expression resulted in a decreased Akt activation downstream from both insulin like growth factor-I (IGF-I) or epidermal growth factor (EGF) stimulation more intensively in MCF7 cells compared to T-47D cells therefore we investigated mitochondrial functions by determining the mitochondrial membrane potential of T-47D and MCF7 derived cells using a MitoProbe™ DilC_1 _[5] assay. As presented in Figure 3 RasGRP3 down-regulation caused a significant decrease in the mitochondrial membrane potencial of MCF7 cells but not in T-47D cells indicating that the protein possibly through the modulation of Akt activation may lead to the activation of the mitochondrial pathway of apoptosis.

In addition, suppression of RasGRP3 expression led to an enhanced sensitivity to the effects of Tamoxifen and Herceptin of the T-47D cell line (but not of MCF7 cells). Of further importance, suppression of RasGRP3 expression reduced xenograft tumor growth and firstly shown in the case of MCF7 cells decreased Ki67 positivity, well-known to be important in molecular classification of breast cancers [41-43]. Collectively, these data strongly suggest that RasGRP3 indeed has a central role in breast cancer tumorigenesis.

**Figure 8:**
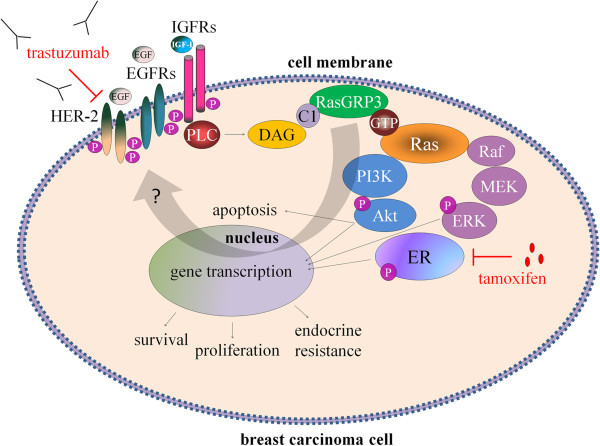
**A schematic overview presenting the role of RasGRP3 in mediating the Ras signaling pathways in breast carcinoma cells.** The signaling of growth factors (such as IGF-I and EGF) through downstream signaling molecules to ERα is mediated by RasGRP3. Inhibition of the Ras signaling pathway at the RasGRP3 level bears the potential to suppress downstream activation and ligand independent activation of ERα resulting in a decreased proliferation and chemotherapeutic resistance. Abbreviations: IGFR: insulin-like growth factor-I receptor; EGFR: epidermal growth factor receptor; HER-2: Human Epidermal Growth Factor Receptor 2; IGF-I: insulin-like growth factor-I; EGF: epidermal growth factor; PLC: phospholipase C; DAG: diacylglycerol; C1: C1 domain; RasGRP3: Ras guanil nucleotid releasing protein 3; GTP: guanosine triphosphate; Ras: H-Ras or R-Ras; Raf: raf kinase; MEK and ERK 1/2: mitogen-activated protein kinases; PI3K: phosphoinositide 3-kinase; Akt: Akt kinase; ER: estrogen receptor alpha; P: phosphate group.

Although RasGRP3 contributes to the signaling of many phospholipase C activating receptors, the focus of studies so far has been on its role in immune cell receptor signaling [44-46]. In the case of the breast, expressions of IGF-I receptor and EGF receptor (EGFR or HER-1) have been reported to be elevated [47-50]. IGF-I and EGF are potent mitogens for breast cancer cells inducing cellular proliferation and promoting the invasiveness and endocrine- or chemotherapeutic resistance of T-47D and MCF7 breast cancer cells [51-55]. Akt and ERK1/2 –downstream targets of Ras- play a critical role in the activation of both cell proliferation and apoptotic signaling and enhance tumor progression by promoting cell invasiveness and angiogenesis [56,57]. Although it was confirmed that modulation of RasGRP3 contributes to Akt and ERK1/2 activation in prostate cancer and melanoma cells [12,13], here we are the first to demonstrate that RasGRP3 contributed to signaling downstream of IGF-I and EGF in the MCF7 and T-47D cells by modifying the level of IGF-I and EGF induced phosphorylation of Akt and ERK 1/2. Namely, down-regulation of RasGRP3 decreased the level of phosphorylated Akt and ERK 1/2 induced by both IGF-I or EGF stimulation in both cell lines, but with a different activation profile. MCF7 derived “RasGRP3-silenced” cells appearing to respond to growth factors induced Akt and ERK1/2 down-regulation earlier and more effectively. The effects of upstream growth factors are mediated in part through RasGRP3 in breast cancer cells; as such these results help fill out the gaps of our current knowledge of the breast cancer signaling network (Figure 8). Furthermore, RasGRP3 not only caused the down regulation of IGF-I and EGF induced downstream signaling, but also decreased the expression of IGF-I and EGF receptors in MCF7 cells. The effects of RasGRP3 on Akt and ERK 1/2 phosphorylation in both T-47D and MCF7 cells are consistent with our demonstration that RasGRP3 is contributing to Ras activation and likewise support the effects we observed on cell proliferation, chemotherapeutic resistance and tumorigenesis. Since it was previously shown that RasGRP3 contributes to carboplatin sensitivity of PC-3 and DU-145 prostate carcinoma cells [12], these results may also be prominently important in the context of endocrine- or chemotherapeutic resistance of breast cancer cells as well.

The primary treatment of ER positive primary and advanced breast cancer is the non-steroidal anti-estrogen tamoxifen, while HER-2/neu positive tumor treatment is focused on trastuzumab, a monoclonal antibody against the HER-2 receptor. Many ER positive tumors are *de novo* resistant to tamoxifen without any prior exposure; furthermore, many of the tumors that initially respond to tamoxifen can aquire resistance during and after tamoxifen therapy which presents a major clinical problem [58-61]. From experimental studies, many different mechanisms have been suggested to explain this resistance, including ligand independent phosphomodification of ERα and an increased crosstalk between ERα and HER-2 receptor [62-64]. Ligand-independent activation of ERα has been reported in response to EGF or IGF-I through ERK1/2, by stimulating the phosphorylation of Ser 118 on the ERα, a clear predictive marker for response to tamoxifen [65,66]. Studies have shown that clinical resistance to tamoxifen can also be associated with EGFR and HER-2 upregulation [67,68]. The tamoxifen resistant MCF7 cell line demonstrates increased EGFR/HER-2/MAPK signaling activity; furthermore, it also shows high levels of phosphorylation of ERα at Ser 118 and enhanced sensitivity to the growth inhibitory actions of EGFR and the HER-2 monoclonal antibody trastuzumab [69]. Taking into account that RasGRP3 is able to regulate the activity of ERK 1/2 and Akt kinases as well as the expression of EGFR, we are the first to investigate the role of RasGRP3 in the development of tamoxifen resistance. Here, we firstly demonstrated the role of RasGRP3 in decreasing the phosphorylation of ERα at Ser 118 through the down-regulation of Akt and ERK 1/2 pathways both in T-47D and MCF7 cells. MCF7 cell line demonstrates higher levels of phosphorylation of ERα at Ser 118 compared to T-47D cells. It can be supposed that in T-47D cells by decreasing ERα transactivation RasGRP3 down-regulation contributed to an increased tamoxifen sensitivity. However, decreasing of ERα activation in MCF7 cells (otherwise more affected cells to EGF and IGF-I treatment) had no effect on tamoxifen sensitivity. Furthermore we demonstrated the role of RasGRP3 in the modulation of the activation of ERα by decreasing the expression of ERα-regulated genes [22], progesterone receptor (PGR), cathepsin D (CTSD) and cytochrome C (CYCS).

Besides tamoxifen we are the first to investigate the role of RasGRP3 in regulation of trastuzumab sensitivity of T-47D and MCF7 cells. The potential mechanisms of resistance to trastuzumab not only involves steric effects, such as the structural mutation in HER2 protein, but the alternative elevations of other tyrosine kinase receptors, such as insulin-like growth factor receptor (IGFIR), or the intracellular alterations in growth factor downstream signaling like constitutive activity of PI3K/Akt pathway [70].These findings imply that cancer cells, by utilizing other growth factor signaling, can compensate for the inhibition of HER2 signaling mediated by trastuzumab. A promising target whose aberrant expression may confer resistance to trastuzumab is the IGF-IR [71] the ectopic expression of IGF-1R drives trastuzumab-sensitive SKBR3 and MCF-7/HER2-18 cells to resistant to the therapy. Although the down regulation of RasGRP3 had no effect on HER-2 expression of the cell lines, in T-47D cells by decreasing the level of phosphorylated Akt and ERK 1/2 induced by IGF-I or EGF stimulation can contribute to an increased sensitivity of the cells against trastuzumab. Since the inhibition of RasGRP3 expression not only caused the down regulation of IGF-I and EGF induced downstream signaling, but also significantly decreased the expression of IGF-I and EGF receptors in MCF7 cells we found it interesting to investigate the role of these receptors in possible role of trastuzumab resistance of MCF7 cells, expressing low amount of HER-2 receptor. According to our results the RasGRP3 gene-silencing and IGFIR down-regulation had no effect on the trastuzumab sensitivity of MCF7 cells.

Collectively, our current findings – i.e. the identification of RasGRP3 as an additional important signaling element together with the evidence for its elevated expression in a subpopulation of breast tumors – identify RasGRP3 as a novel, promising target molecule for pathway directed chemotherapy in the supportive treatment and diagnosis of ductal adenocarcinoma of human breast.

## Conclusions

Breast cancer is the leading cause of cancer death among females. Several studies have identified the expression of RasGRP3 on different malignant cancer types, but in this study we show for the first time that RasGRP3 is overexpressed in human breast cancer samples as well as in multiple breast cancer derived cell lines. Our *in vitro* and *in vivo* data, together with that of others [12,13] have shown the importance of RasGRP3 function in the regulation of proliferation, survival, chemotherapeutic sensitivity and tumorigenesis of breast carcinoma cells. We have further demonstrated that RasGRP3 contributed to Ras-related signaling downstream of IGF-I and EGF growth factors by modifying the level of IGF-I and EGF induced activation of Akt, ERK ½ kinases, estrogen receptor α and the expression of IGF-I and EGF growth factor receptors. Collectively, these data suggest that the overexpression of the protein might be a relatively early step in the process of tumorigenesis; hence, the determination of RasGRP3 levels in breast cancer tissues may hold promise for the benefits of early diagnosis and may offer suitable target for treatment of breast tumors.

## Materials and methods

### Human tissues

The human study was approved by the Institutional Research Ethics Committee of the University of Debrecen, Debrecen, Hungary and by various authorities of the Hungarian Government. All patients provided written informed consent for participation in the study and publication of the results. Twenty-one normal adult (healthy) breast tissue samples and 33 breast derived ductal adenocarcinoma samples (grade I-III) were obtained as part of routine diagnosis from surgical specimen. Samples were verified by histopathological evaluations by expert pathologists. Neither the control patients nor the diseased patients had history of previous or contemporary breast malignancies.

### Human tissue sample preparation

The tumor samples were divided into two parts. One part of the samples was processed to obtain formalin-fixed, paraffin-embedded sections which were subjected to routine haematoxylin-eosin staining-based grading according to the Nottingham scale (the pathohistological diagnosis of the individual tumors is detailed in Figure 1A and B). The second part was frozen in liquid nitrogen and processed for Q-PCR and Western blot analysis (see below).

### Antibodies for Western blotting and immunolabeling

Throughout the experiments, the following primary antibodies were used: RasGRP3 and phospho-RasGRP3 (Thr133); p44/42 MAP kinase (ERK 1/2) and phospho-p44/42 MAP kinase (phospho-ERK 1/2); Akt and phospho-Akt (Ser473) antibodies for Western blot experiments were obtained from Cell Signaling Technology (Beverly, MA). Anti-RasGRP3 antibody for immunohystochemistry was purchased from Abcam (Cambridge, UK). Ki67 antibody was obtained from DAKO (Glostrup, Denmark). Actin β, ERα, p-ERα (Ser 118) and HER-2 antibodies were purchased from Sigma-Aldrich (St. Louis, MO). IGF-IR and EGFR antibodies were obtained from Santa Cruz Biotechnology (Dallas, TX). Glyceraldehyde 3-phosphate dehydrogenase (GAPDH) antibody was obtained from Novus Biologicals (Cambridge, UK). Appropriate secondary antibodies were purchased from Bio-Rad Laboratories (Hercules, CA).

### Immunohistochemistry

The expression of RasGRP3 and phosphoRasGRP3 were determined by a horseradish-peroxidase (HRP) based method with diaminobenzidine (DAB) as a chromogene. Tissues were fixed overnight in 4% paraformaldehyde, followed by paraffin infiltration and embedding. Sections were de-waxed and subjected to heat-induced epitope retrieval in 10 mM citrate buffer (pH 6.0) at 750 W in microwave oven for 10 min prior to treatment with 3% hydrogen peroxide in absolute methanol. Sections were then incubated in blocking buffer containing 0.6% Triton X-100 and 1% bovine serum albumin (all from Sigma-Aldrich) for 30 min and probed with the appropriate primary antibodies. Sections were then stained for 30 minutes with the appropriate horseradish peroxidase-labeled polymer conjugated secondary antibodies. Immunoreactions were visualized using DAB substrate (EnVision kit, DAKO)) for 3–5 minutes and the sections were counterstained by Mayer’s hematoxylin (Sigma-Aldrich) and cover-slipped for microscopic examination. To assess specificity of the immunostaining antibodies were either omitted from the procedure or were pre-absorbed by control blocking peptides provided (along with appropriate protocols) by the manufacturers. Images of RasGRP3 and phospho-RasGRP3-labeled sections were photographed at 20x magnification (see Figure 1C). Immunohistochemical images were captured and digitalized using an RT Spot Colour CCD camera (Diagnostic Instruments Inc., Sterling Heights, MI) integrated on a Nikon Eclipse 600 fluorescence and light microscope (Nikon, Tokyo, Japan).

### Cell culturing

In this study, we employed six different breast derived ductal adenocarcinoma cell lines (BT-474, MDA-MB-453, MCF7, SK-BR-3, JIMT-1, T-47D) and, as a positive control [12], a prostate derived carcinoma cell line (PC-3). Human breast cancer cell lines were kind gifts from Dr. Péter Nagy at the University of Debrecen, Medical and Health Science Center Faculty of Medicine, Department of Biophysics and Cell Biology, Debrecen, Hungary. PC-3 cell line was kindly provided by the Laboratory for Cancer Biology and Genetics, National Cancer Institute, National Institute of Health, Bethesda, Maryland, USA. PC-3, BT-474 and T-47D cells were maintained in RPMI-1640 Medium (Sigma-Aldrich), MCF7 cells were maintained in Eagle's Minimum Essential Medium (MEM) (Sigma-Aldrich), JIMT-1 cells were maintained in Dulbecco’s Modified Eagle Medium (DMEM)/Ham’s F12 Medium (Sigma-Aldrich), SK-BR-3 cells were maintained in Dulbecco’s Modified Eagle Medium (DMEM) and MDA-MB-453 cells were maintained in Leibovitz’s 15 Medium (Invitrogen). All medium were supplemented with 10% (v/v) fetal bovine serum (FBS, Invitrogen, Paisley, UK), 2 mM Glutamine (Sigma-Aldrich), 50 U/ml penicillin and 50 μg/ml streptomycin (both from TEVA, Debrecen, Hungary). In addition, medium for T47-D cells was supplemented with 0.2 U/ml bovine insulin (Sigma-Aldrich), medium for MCF7 cells was supplemented with 0.01 mg/ml bovine insulin and medium for JIMT-1 cells was supplemented with 60 U/l bovine insulin. Medium was changed every other day and cells were sub-cultured at 80% confluence at 37°C in a humidified atmosphere with 5% CO_2_.

### Western blotting

Tissues and cells were homogenized in lysis buffer (20 mM Tris-Cl, 5 mM EGTA, pH 7.5 and protease inhibitor cocktail all from Sigma-Aldrich) and the protein content of samples was measured by the BCA protein assay kit (Pierce, Rockford, IL). The lysates containing 20 μg total protein (except for RasGRP3 and phosphoRasGRP3 immunoblotting where 60 μg total protein was used) were separated by electrophoresis on 10% SDS-polyacrylamide gels (Invitrogen) and transferred onto BioBond nitrocellulose membranes (Whatman, Maidstone, UK). After the membranes were blocked and labeled with the appropriate primary and secondary antibodies, the immunoreactive bands were visualized by SuperSignal West Femto Chemiluminescent Substrate-enhanced chemiluminescence (Pierce, Rockford, IL) using a Gel Logic 1500 Imaging System (Kodak, Tokyo, Japan). To assess equal loading (and to obtain an endogenous control), membranes were re-probed with a GAPDH or actin β antibody followed by a similar visualization procedure as described above.

### Quantitative real-time PCR (Q-PCR)

Quantitative real-time PCR was performed on an ABI Prism 7000 sequence detection system (Applied Biosystems, Foster City, CA) by using the 5′ nuclease assay, as we have previously described [72]. Total RNA was extracted with TRIzol reagent (Invitrogen) according to manufacturer's protocol. One microgram of total RNA were then reverse transcribed into cDNA by using High Capacity cDNA Reverse Transcription Kit (Applied Biosystems) according to the manufacturer’s instructions. PCR amplification was performed by using the TaqMan primers and probes (Assay IDs: Hs 00964396_m1 for human RasGRP3; Hs 01076078_m1 for human EGFR; Hs 00609566_m1 for human IGF1R; Hs 01001580_m1 for human HER-2/ERBB2; Hs 01894962_s1 for human LOR; Hs01588974_g1 for human CYCS; Hs 00157205_m1 for humanCTSD ans Hs 01556702 for human PGR) using the TaqMan Universal PCR Master Mix Protocol (Applied Biosystems). The threshold cycle (Ct) of RasGRP3 was determined and normalized to that of human GAPDH (Assay ID: Hs 03929097_g1) to obtain a ΔCt value (Ct_GAPDH_-Ct_appropriate protein_) from each sample. The Q-PCR was performed in triplicate.

### shRNA construct for RasGRP3

Gene knockdown was achieved by stable transduction with a retroviral-based pRNA-H1.1/Retro Vector system (GenScript, Piscataway, NJ) containing previously described RasGRP3-specific hairpin RNA (shRasGRP3; sequence listed in Additional file 1: Table S1) os scrambled control sequence (shSCR; sequence listed in Additional file 1: Table S1) which has no specific targets in mammalian cells [12,13]. The designed single-stranded oligonucleotides were synthesized, annealed, inserted into the pRNA-H1.1/Retro vector by GenScript. All constructs were verified by DNA sequencing. The retroviral constructs were then produced and titered by following the manufacturer’s instructions (Clontech, CA). The two host cell lines, MCF7 and T-47D were infected with each retroviral supernatant and subjected to Hygromycin B (400 μg/ml) selection for 3 weeks. Studies were carried out on the clone selected, antibiotic-resistant cells. Efficiency of the suppression of RasGRP3 expression was determined using Western blotting as described above.

### Cell proliferation assay

The degree of cellular growth was determined by measuring the DNA content of cells using CyQUANT GR Cell Proliferation Assay Kit (Invitrogen) according to the manufacturer's protocol. MCF7 and T-47D cells (10 000 cells per well) were cultured in 96-well black-well/clear-bottom plates (Greiner Bio-One, Baden-Württemberg, Germany) in quadruplicates and were growth for 5 days. The assay was performed each day. Supernatants were removed by blotting on paper towels, and the plates were subsequently frozen at -70°C. The plates were then thawed at room temperature, and 200 μl of CyQUANT GR dye/cell lysis buffer mixture was added to each well. After 5 minutes of incubation, fluorescence signals were quantitated on a Fluorescence Imaging Plate Reader FlexStation^III^ (FLIPR, Molecular Devices, CA) at an excitation wavelength of 485 nm and an emission wavelength of 530 nm [73].

The CyQUANT assay was employed to assess the chemotherapeutic sensitivity of the cells. For this, MCF7 and T-47D cells were plated in 96-well black-well/clear-bottom plates (Greiner Bio-One) in quadruplicates at a density of 10^4^ cells/well*.* After overnight culturing, various concentrations of chemotherapeutics, i.e. trastuzumab (F.Hoffmann-LaRoche Ltd., Switzerland) and tamoxifen (Sigma-Aldrich) were administered for 72 h. After the indicated time, cells were suspended in 200 μl CyQuant GR cell proliferation assay binding solution and analyzed as described above.

### Detection of apoptotic and necrotic cells

Cells were harvested, washed once in ice-cold Dulbecco’s phosphate buffered saline (DPBS), and re-suspended in 0.5 ml DPBS. Annexin-V (Invitrogen) was added to a final concentration of 1 μM and the cells were incubated for 20 min at 4°C in the dark. Propidium iodide (PI) (Invitrogen) was then added at a 5 μg/ml final concentration 10 minutes before analysis by flow cytometry using the FACScan system (BD Biosciences, Oxford, UK). Data were analyzed with FSC Express software (De Novo Softwer, CA).

Mitochondrial membrane potential was also assessed by MitoProbe DilC_1_(5) Assay Kit (Invitrogen). T-47D and MCF7 derived cells (5,000 cells/well) were cultured in 96-well black-well/clear-bottom plates in quadruplicate. After removal of supernatants, cells were incubated with DilC_1_(5) working solution –prepared according to the manufacturer’s instructions- and the fluorescence of DilC_1_(5) was measured at 630-nm excitation and 670-nm emission wavelengths using Fluorescence Imaging Plate Reader FlexStation^III^.

### Xenograft experiments

Severe combined immunodeficiency (SCID) mice of 12–13 weeks of age were provided by Dr. György Vereb (University of Debrecen, Medical and Health Science Center Faculty of Medicine, Department of Biophysics and Cell Biology, Debrecen, Hungary). The mice were bred and maintained in the animal facility of the Department of Dermatology (University of Debrecen, Medical and Health Science Center Faculty of Medicine) in accordance with the animal-welfare ordinance. The studies were performed under the current regulations and standards of the Institutional Research Ethics Committee of the University of Debrecen, Debrecen, Hungary. Cells were harvested by trypsinization and washed twice with DPBS. Cell pellets (4× 10^6^ cells) were re-suspended in 0.1 ml appropriate medium and 0.1 ml Matrigel (BD Biosciences) and injected in a single subcutaneously site on the right flank of the mice (0.2 ml/injection). The animals were sacrificed after 8 weeks and the tumors were excised, weighed and placed in 4% paraformaldehyde for immunohistochemical analysis. To evaluate proliferation Ki67-specific labeling was performed according to the manufacturer instructions (see Figure 5B and Additional file 3: Figure S1 and Additional file 4: Figure S2). The histopathological evaluations of the xenograft tumor derived sections were verified by expert pathologists.

### Image analysis

Immunohistochemical images were captured and digitalized using an RT Spot Colour CCD camera (Diagnostic Instruments Inc.) integrated on a Nikon Eclipse 600 fluorescence and light microscope (Nikon). Digitalized images were then analyzed using Image J (NIH, Bethesda, MD) image analysis software. The number of cells were counted at five randomly placed, equal areas of interest (AOI) and the average of Ki67 imunnopositive cells of the 5 AOI was defined.

### Statistical analysis

The data are expressed as mean +/-SEM. Significance differences were assessed by a two-tailed un-paired *t-*test (p < 0.05 values were defined as significance). The symbol of * indicates significant difference compared to scrambled control cells, except if it is indicated otherwise.

## Abbreviations

RasGEF: Guanine nucleotide exchange factors; RasGRP3: Ras Guanyl nucleotide Releasing Peptide 3; DAG: diacylglycerol; C1: C1 domain; shRasGRP3: RasGRP3-specific shRNA; shSCR: Scrambled control shRNA; PI: Propidium jodide; SCID: Severe combined immunodeficiency; IGFR: Insulin-like growth factor-I receptor; EGFR: Epidermal growth factor receptor; HER-2/ERBB2: Human Epidermal Growth Factor Receptor 2; IGF-I: Insulin-like growth factor-I; EGF: Epidermal growth factor; Ras: H-Ras or R-Ras; MEK and ERK 1/2: Mitogen-activated protein kinases; Akt: Akt kinase; ERα: Estrogen receptor alpha; PGR: Progesterone receptor; CTSD: Cathepsin D; LOR: Loricrin; CYCS: Cytochrome C; p: Phosphorilated forms.

## Competing interests

No potential conflicts of interest were disclosed.

## Authors’ contributions

ZN conceived of the hypothesis, designed and performed the experiments, analyzed data and wrote the manuscript. IK conducted pathology review of the histopathology slides, independently graded and typed the tumors. MT conducted pathology review of the histopathology slides, independently graded and typed the tumors, identified and collected samples and provided assistance with the *in vivo* experimentation. DT identified and collected samples and provided clinical details. GV provided the SCID mice and managed *in vivo* experimentation. KB provided packaging cell line PT67. IJ managed *in vivo* experimentation. PB conceived of the hypothesis, provided material and intellectual support. TB and CG conceived of the hypothesis, contributed to obtaining all necessary approvals and clearances to conduct the research, contributed to obtaining grant funding, supervised aspects of the research, co-wrote the manuscript and approved the final version. All authors revised the manuscript and gave their final approval.

## Supplementary Material

Additional file 1: Table S1 Characteristics of the cell lines used in the study. Click here for file

Additional file 2: Table S2 shRNA sequences of shRasGRP3 and non-targeting scrambled control. Click here for file

Additional file 3: Figure S1 Inhibition of RasGRP3 expression inhibites xenograft tumor growth of T-47D cells. (A) Representative images of T-47D xenografts (n=4) injected with shSCR or shRasGRP3 derived cells taken during dissection. Black arrows indicate the tumors. Scale bar: 10 mm. (B) Haematoxylin-eosin stained representative images of the developed shSCR or shRasGRP3 xenograft tumors. T-47D cells derived tumors showed „ Pushing-type” of growth reflected in an infiltrative and expansive growth pattern with mechanical pressure to the sorrounded tissues. Necrotic areas (NeA) frequently developed in these tumors. Scale bar: 200 μm. (C) Representative images of Ki67-specific immunoreactivity with diaminobenzidine as a chromogen (brown staining) on sections prepared from tumors developed by shSCR or shRasGRP3 derived cells. Nuclei were co-stained by Mayer’s Hematoxylin (blue staining). Scale bar: 100 μm. Click here for file

Additional file 4: Figure S2 Inhibition of RasGRP3 expression inhibites xenograft tumor growth of MCF7 cells. (A) Representative images of MCF7 xenografts (n=4) injected with shSCR or shRasGRP3 derived cells taken during dissection. Black arrows indicate the tumors. Scale bar: 10 mm. (B) Haematoxylin-eosin stained representative images of the developed shSCR or shRasGRP3 xenograft tumors. Compared to T-47D cells MCF7 derived tumors are composed of more differentiated tumor tissue with less infiltrative nature. In these tumors no large necrotic areas were present. Scale bar : 200 μm. (C) Representative images of Ki67-specific immunoreactivity with diaminobenzidine as a chromogen (brown staining) on sections prepared from tumors developed by shSCR or shRasGRP3 derived cells. Nuclei were co-stained by Mayer’s Hematoxylin (blue staining). Scale bar: 100 μm. Click here for file

Additional file 5: Figure S3 Effects of down-regulation of RasGRP3 on the Ras signaling pathway I. RasGRP3 is involved in IGF-I dependent Akt, ERK and ERα activation. RasGRP3 knockdown cell lines created from T-47D (A) and MCF7 (B) cells were treated with or without IGF-I (100 pg/ml) as indicated. Akt and phosphorylated Akt, ERK and phosphorylated ERK, ERα and phosphorylated ERα were detected by immunoblotting of cell lysates. Levels of total Akt, ERK and ERα were used as control. All results were representative of 2 independent experiments. Click here for file

Additional file 6: Figure S4 Effects of down-regulation of RasGRP3 on the Ras signaling pathway II. RasGRP3 is involved in EGF dependent Akt, ERK and ERα activation. RasGRP3 knockdown cell lines created from T-47D (A) and MCF7 (B) cells were treated with or without EGF (100 pg/ml) as indicated. Akt and phosphorylated Akt, ERK and phosphorylated ERK, ERα and phosphorylated ERα were detected by immunoblotting of cell lysates. Levels of total Akt, ERK and ERα were used as control. All results were representative of 2 independent experiments. Click here for file
