# Supplementary material for: Function of RasGRP3 in the formation and progression of human breast cancer
Source: Mol Cancer. 2014 Apr 29;13:96. doi: 10.1186/1476-4598-13-96 (PMC4113147; doi:10.1186/1476-4598-13-96)
Supplement: Additional file 1: Table S1 — Characteristics of the cell lines used in the study. [file 1476-4598-13-96-S1.docx]

| **Cell line** | **estrogen receptor** | **tamoxifen resistant** | **trastuzumab resistant** | **primary** | **metastatic**  **(from)** | **HER-2 receptor** |
| --- | --- | --- | --- | --- | --- | --- |
| BT-474 | expressed | no | no | yes |  | expressed |
| JIMT-1 | not expressed | yes | yes |  | yes  (pleura) | overexpressed |
| MCF7 | expressed | no | no |  | yes  (pleura) | not  expressed |
| SK-BR-3 | not expressed | no | no |  | yes  (pleura) | expressed |
| MDA-MB-453 | not expressed | no | no |  | yes  (pericardium) | expressed |
| T-47D | expressed | no | no |  | yes  (pleura) | expressed |

**Supplementary Table 1.** Characteristics of the cell lines used in the study.

The statements are supported by the ATCC, the following references as well as our experiments.

1. Neve RM, Chin K, Fridlyand J, Yeh J, Baehner FL, Fevr T, Clark L, Bayani N, Coppe JP, Tong F, Speed T, Spellman PT, DeVries S, Lapuk A, Wang NJ, Kuo WL, Stilwell JL, Pinkel D, Albertson DG, Waldman FM, McCormick F, Dickson RB, Johnson MD, Lippman M, Ethier S, Gazdar A, Gray JW. **A collection of breast cancer cell lines for the study of functionally distinct cancer subtypes**. *Cancer Cell* 2006; **10**:515–527.
2. Prat A, Parker JS, Karginova O, Fan C, Livasy C, Herschkowitz JI, He X, Perou CM. **Phenotypic and molecular characterization of the claudin-low intrinsic subtype of breast cancer**. *Breast Cancer Res* 2010; **12**:R68.
3. [Tanner M](http://www.ncbi.nlm.nih.gov/pubmed?term=Tanner%20M%5BAuthor%5D&cauthor=true&cauthor_uid=15634652), [Kapanen AI](http://www.ncbi.nlm.nih.gov/pubmed?term=Kapanen%20AI%5BAuthor%5D&cauthor=true&cauthor_uid=15634652), [Junttila T](http://www.ncbi.nlm.nih.gov/pubmed?term=Junttila%20T%5BAuthor%5D&cauthor=true&cauthor_uid=15634652), [Raheem O](http://www.ncbi.nlm.nih.gov/pubmed?term=Raheem%20O%5BAuthor%5D&cauthor=true&cauthor_uid=15634652), [Grenman S](http://www.ncbi.nlm.nih.gov/pubmed?term=Grenman%20S%5BAuthor%5D&cauthor=true&cauthor_uid=15634652), [Elo J](http://www.ncbi.nlm.nih.gov/pubmed?term=Elo%20J%5BAuthor%5D&cauthor=true&cauthor_uid=15634652), [Elenius K](http://www.ncbi.nlm.nih.gov/pubmed?term=Elenius%20K%5BAuthor%5D&cauthor=true&cauthor_uid=15634652), [Isola J](http://www.ncbi.nlm.nih.gov/pubmed?term=Isola%20J%5BAuthor%5D&cauthor=true&cauthor_uid=15634652). **Characterization of a novel cell line established from a patient with Herceptin-resistant breast cancer.** [*Mol Cancer Ther*.](http://www.ncbi.nlm.nih.gov/pubmed/15634652) 2004; **3**:1585-92.
