# Supplementary material for: Function of RasGRP3 in the formation and progression of human breast cancer
Source: Mol Cancer. 2014 Apr 29;13:96. doi: 10.1186/1476-4598-13-96 (PMC4113147; doi:10.1186/1476-4598-13-96)
Supplement: Additional file 2: Table S2 — shRNA sequences of shRasGRP3 and non-targeting scrambled control. [file 1476-4598-13-96-S2.docx]

| shRNA sequences | |
| --- | --- |
| **shRasGRP3** | ACGCGTCGCAATTACCGCAAGGCCTTTGTTGATATCCGCAAAGGCCTTGCGGTAATTGCTTTTTTCCAACTCGAG |
| **shSCR** | ACGCGTCGATAGCATTCGGACGTTGCTATTGATATCCGTAGCAACGTCCGAATGCTATCTTTTTTCCAACTCGAG |

**Supplementary Table 2.** shRNA sequences of shRasGRP3 and non-targeting scrambled control.
