# Supplementary figures and images for: Function of RasGRP3 in the formation and progression of human breast cancer
Source: Mol Cancer. 2014 Apr 29;13:96. doi: 10.1186/1476-4598-13-96 (PMC4113147; doi:10.1186/1476-4598-13-96)

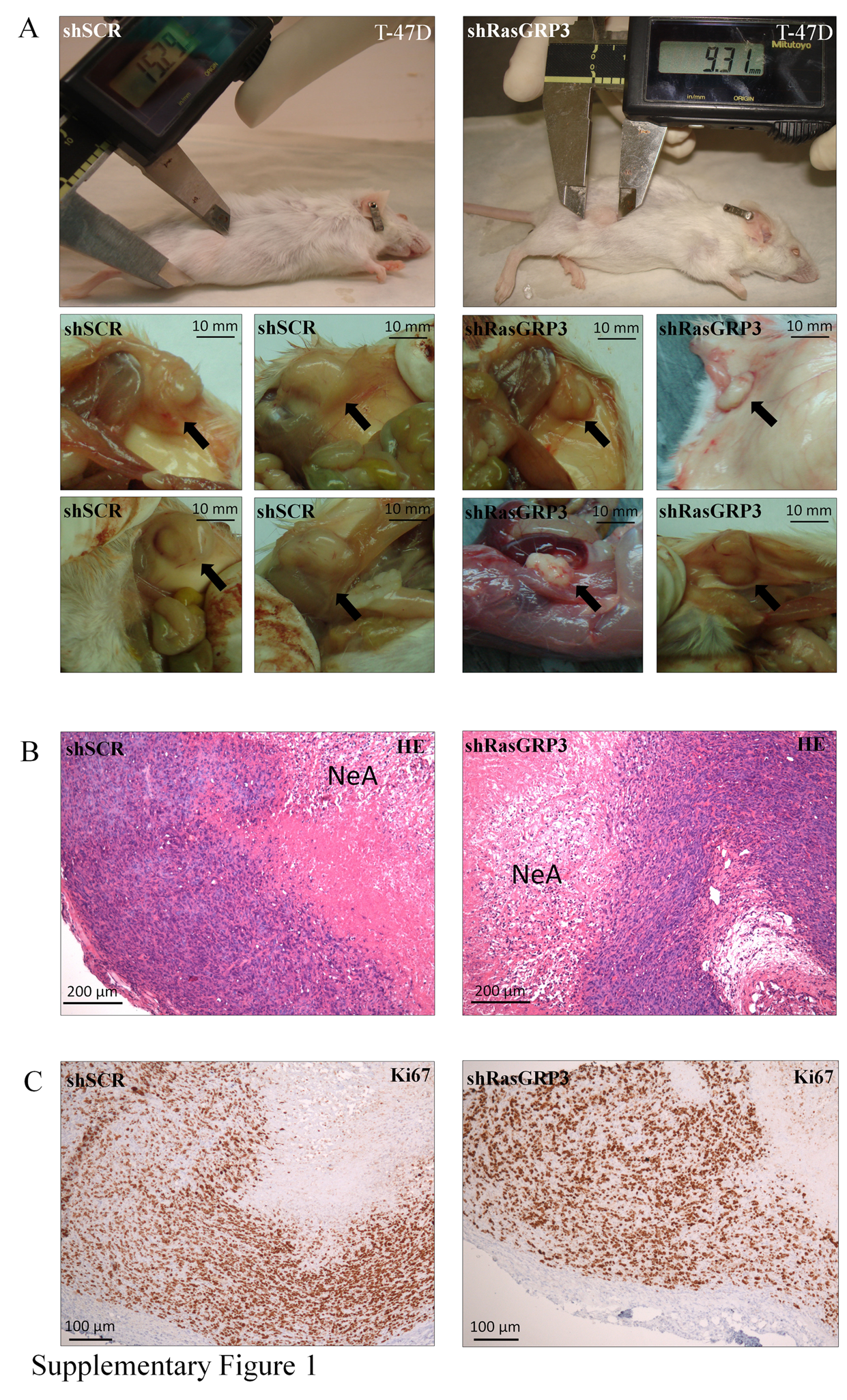

Supplement: Additional file 3: Figure S1 — Inhibition of RasGRP3 expression inhibites xenograft tumor growth of T-47D cells. (A) Representative images of T-47D xenografts (n=4) injected with shSCR or shRasGRP3 derived cells taken during dissection. Black arrows indicate the tumors. Scale bar: 10 mm. (B) Haematoxylin-eosin stained representative images of the developed shSCR or shRasGRP3 xenograft tumors. T-47D cells derived tumors showed „ Pushing-type” of growth reflected in an infiltrative and expansive growth pattern with mechanical pressure to the sorrounded tissues. Necrotic areas (NeA) frequently developed in these tumors. Scale bar: 200 μm. (C) Representative images of Ki67-specific immunoreactivity with diaminobenzidine as a chromogen (brown staining) on sections prepared from tumors developed by shSCR or shRasGRP3 derived cells. Nuclei were co-stained by Mayer’s Hematoxylin (blue staining). Scale bar: 100 μm. [file 1476-4598-13-96-S3.tiff]

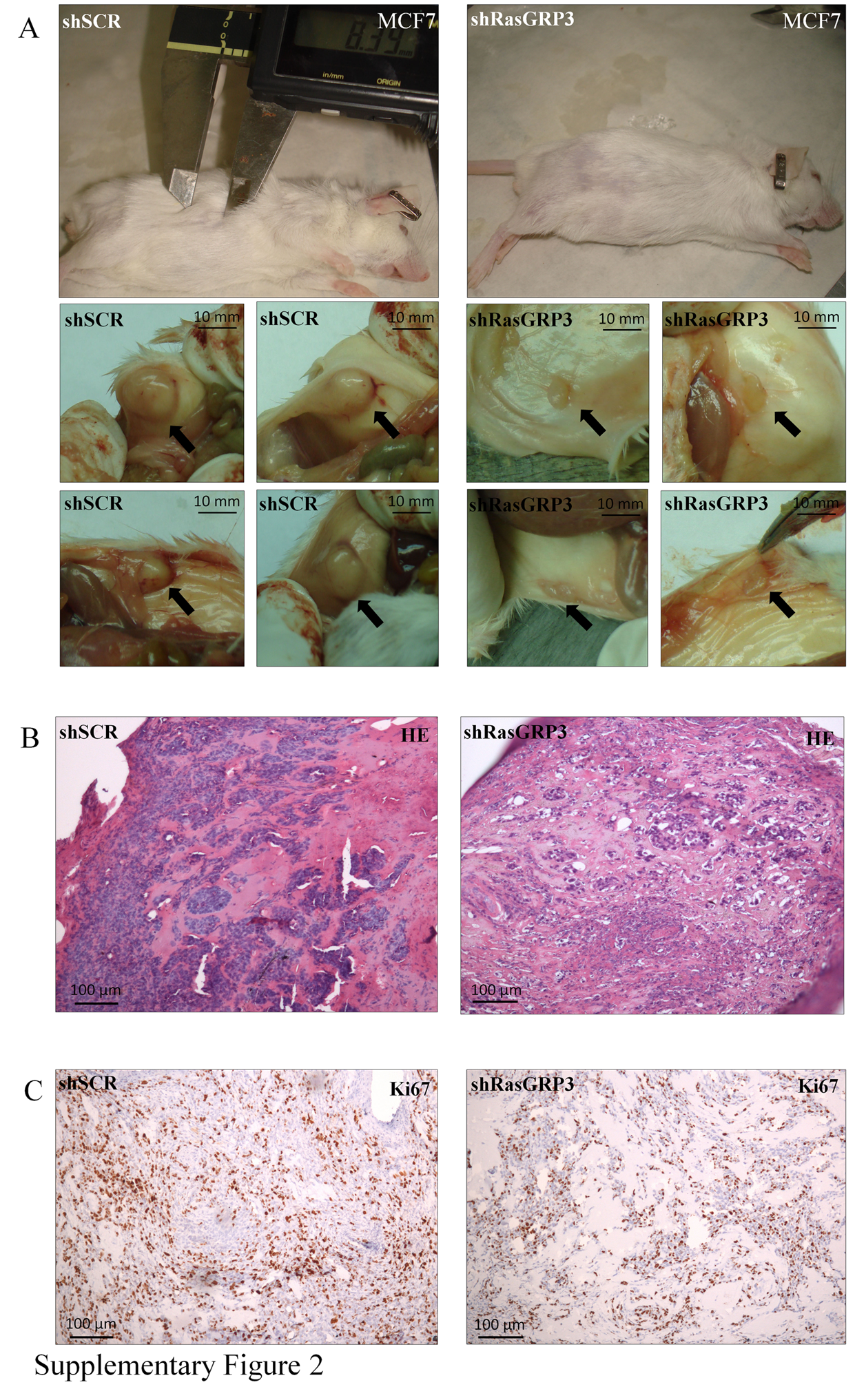

Supplement: Additional file 4: Figure S2 — Inhibition of RasGRP3 expression inhibites xenograft tumor growth of MCF7 cells. (A) Representative images of MCF7 xenografts (n=4) injected with shSCR or shRasGRP3 derived cells taken during dissection. Black arrows indicate the tumors. Scale bar: 10 mm. (B) Haematoxylin-eosin stained representative images of the developed shSCR or shRasGRP3 xenograft tumors. Compared to T-47D cells MCF7 derived tumors are composed of more differentiated tumor tissue with less infiltrative nature. In these tumors no large necrotic areas were present. Scale bar : 200 μm. (C) Representative images of Ki67-specific immunoreactivity with diaminobenzidine as a chromogen (brown staining) on sections prepared from tumors developed by shSCR or shRasGRP3 derived cells. Nuclei were co-stained by Mayer’s Hematoxylin (blue staining). Scale bar: 100 μm. [file 1476-4598-13-96-S4.tiff]

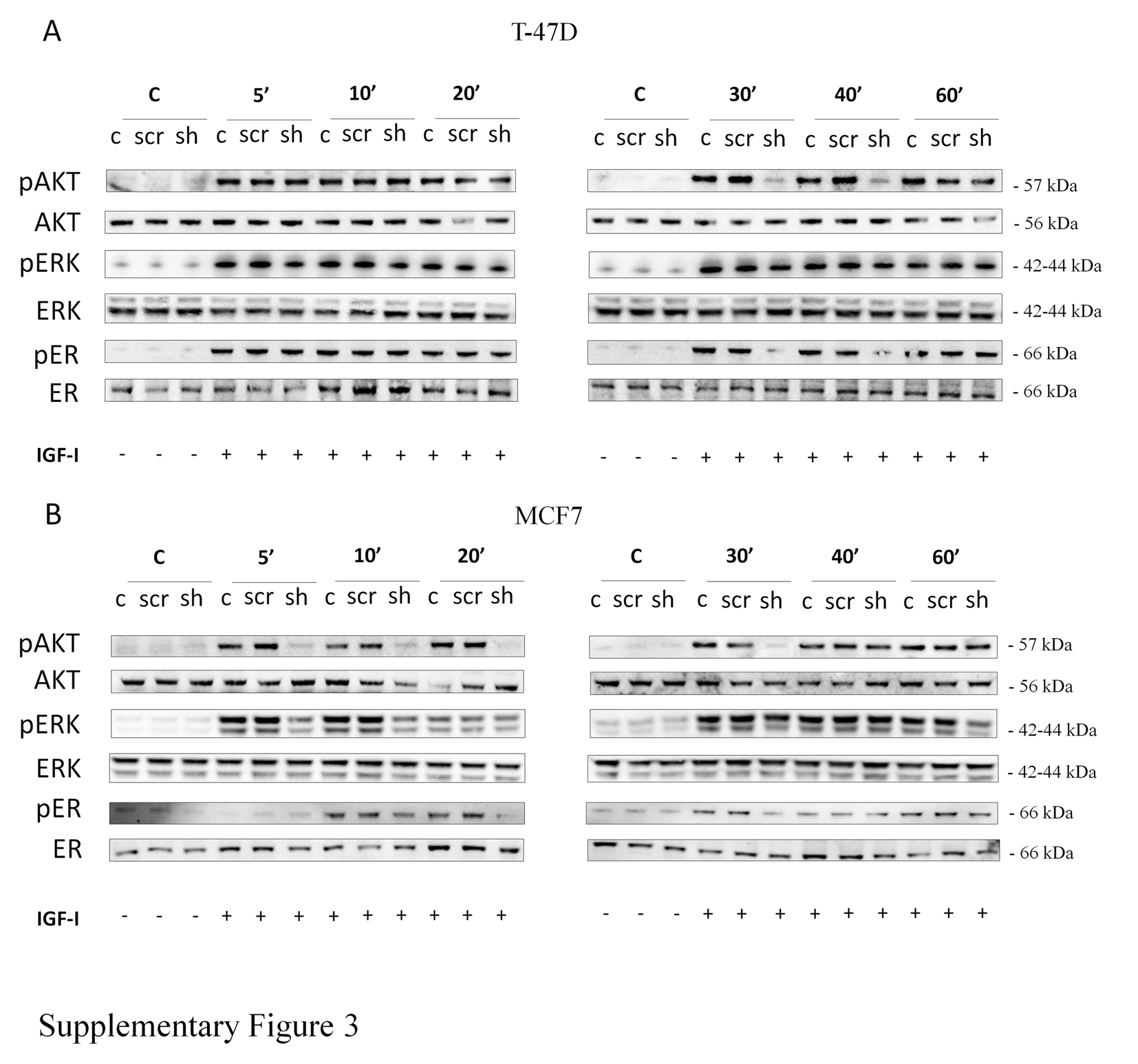

Supplement: Additional file 5: Figure S3 — Effects of down-regulation of RasGRP3 on the Ras signaling pathway I. RasGRP3 is involved in IGF-I dependent Akt, ERK and ERα activation. RasGRP3 knockdown cell lines created from T-47D (A) and MCF7 (B) cells were treated with or without IGF-I (100 pg/ml) as indicated. Akt and phosphorylated Akt, ERK and phosphorylated ERK, ERα and phosphorylated ERα were detected by immunoblotting of cell lysates. Levels of total Akt, ERK and ERα were used as control. All results were representative of 2 independent experiments. [file 1476-4598-13-96-S5.tiff]

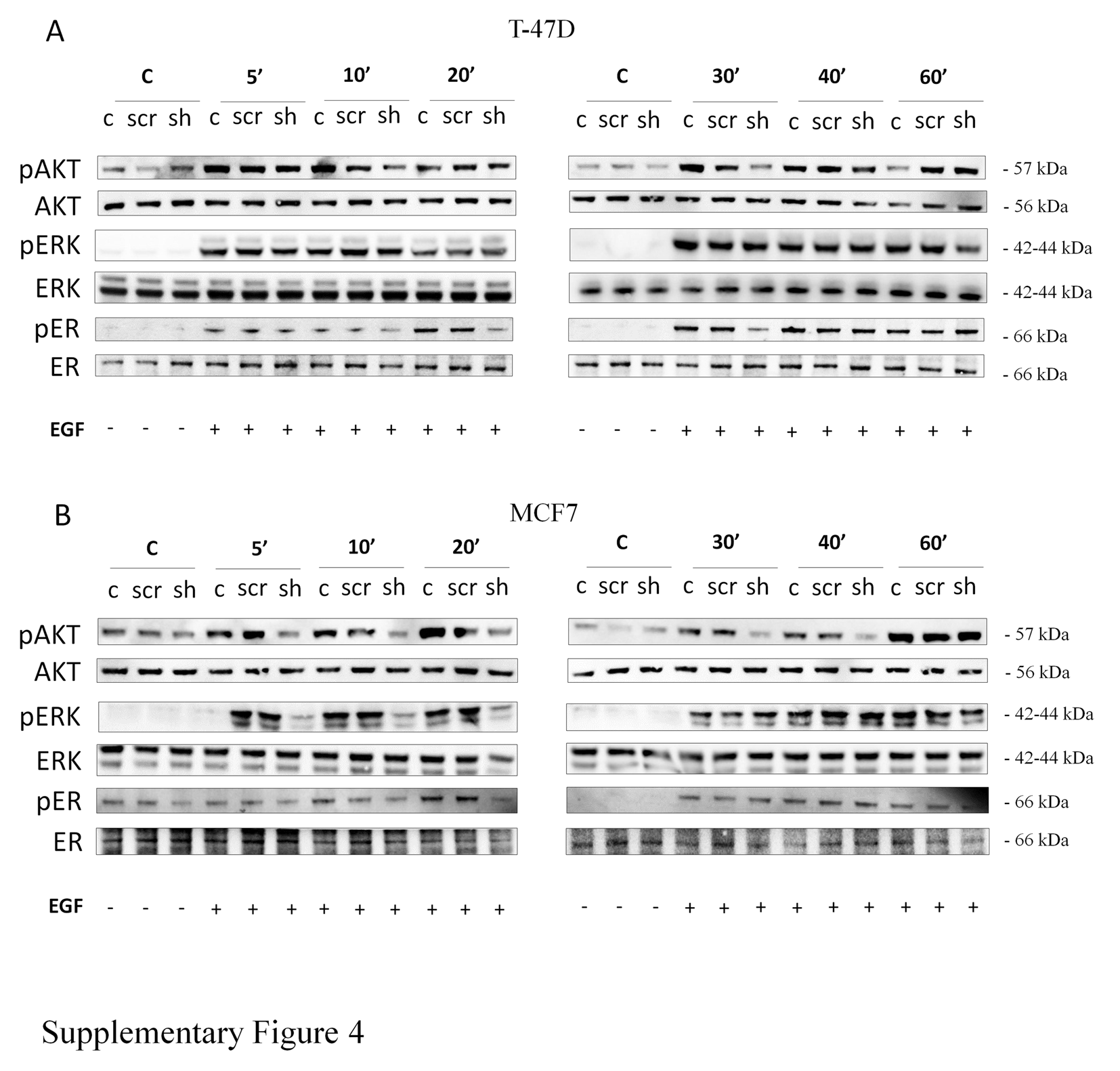

Supplement: Additional file 6: Figure S4 — Effects of down-regulation of RasGRP3 on the Ras signaling pathway II. RasGRP3 is involved in EGF dependent Akt, ERK and ERα activation. RasGRP3 knockdown cell lines created from T-47D (A) and MCF7 (B) cells were treated with or without EGF (100 pg/ml) as indicated. Akt and phosphorylated Akt, ERK and phosphorylated ERK, ERα and phosphorylated ERα were detected by immunoblotting of cell lysates. Levels of total Akt, ERK and ERα were used as control. All results were representative of 2 independent experiments. [file 1476-4598-13-96-S6.tiff]
